# Dam Safety Evaluation Based on Interval-Valued Intuitionistic Fuzzy Sets and Evidence Theory

**DOI:** 10.3390/s20092648

**Published:** 2020-05-06

**Authors:** Xiaosong Shu, Tengfei Bao, Yangtao Li, Kang Zhang, Bangbin Wu

**Affiliations:** 1 State Key Laboratory of Hydrology-Water Resources and Hydraulic Engineering, Hohai University, Nanjing 210098, China; 180802020004@hhu.edu.cn (X.S.); liyangtao@hhu.edu.cn (Y.L.); zhangkang@hhu.edu.cn (K.Z.); wubangbin1986@126.com (B.W.); 2 College of Water-conservancy and Hydropower, Hohai University, Nanjing 210098, China; 3 College of Hydraulic & Environmental Engineering, China Three Gorges University, Yichang 443002, China

**Keywords:** dam engineering, evidence theory, interval-valued intuitionistic fuzzy set, multi-source information fusion

## Abstract

Considering the multi-sources, heterogeneity and complexity of dam safety assessment, a dam safety assessment model based on interval-valued intuitionistic fuzzy set and evidence theory is proposed to perform dam safety reliability evaluations. In the proposed model, the dynamic reliability based on the supporting degree is applied to modify the data from homologous information. The interval-valued intuitionistic fuzzy set is used to describing the uncertainty and fuzziness between heterogeneous information. Evidence theory is employed to integrate the data from heterogeneous information. Finally, a multiple-arch dam undergoing structural reinforcement is taken as an example. The evaluation result before reinforcement shows that the safety degree of the dam is low and the potential risk is more likely to be located at the dam section #13. From the geological survey before reinforcement, there exist weak fracture zone and broken mud belt in the foundation of the dam section #13. The comparison between the evaluation results before and after reinforcement indicates that the dam become safer and more stable after reinforcement.

## 1. Introduction

In the early 21th century, there exist about 98,000 dams in active service in China. Forty percent of these dams are facing safety problems due to low design standards, poor construction quality, serious aging, and other hidden factors. With the increase of service life, more and more dams will have the same problems [1]. Therefore, it is very important to diagnose the dam behavior accurately, obtain the safety degree, and identify potential risks for proper reinforcement at the right time.

There are many factors affecting the performance of dam behavior, such as water levels, temperature, concrete deterioration, etc. [2]. Several features are monitored to reflect the working condition of dams during the operation period, like deformation, seepage, crack width, etc. Therefore, the safety assessment of the dam behavior is a complex multisource data fusion problem. In the multisource data system of a dam, the information derived from different sources is usually imperfect, imprecise, uncertain, or even conflicting. Therefore, it is difficult to make use of the information directly. 

In recent years, uncertainty theories such as probability theory, evidence theory, fuzzy set theory, and possibility theory have been proposed to handle the problem of multisource data fusion [3]. Among these theories, Dempster-Shafer evidence theory (DST) has been widely applied in multisource information fusion because of its flexibility in managing uncertainty. Basir et al. [4] applied DST as a tool for fusing multi-sensory evidence pertinent to engine quality. Ran et al. [5] presented a decision-fuse method to provide a higher-accuracy land cover map by combining multisource data based on DST. Xiao [6] proposed a new method for multi-sensor data fusion based on a new belief divergence measure of evidences and the belief entropy. Zhang et al. [7] proposed a hybrid multi-attribute decision making method by combining the DST and data envelopment analysis. From the literature mentioned above, DST is a recursive theory based on decision theory and evidence theory, which performs well in handling the fuzzy data fusion and solving fuzzy decision-making problem. Considering the incompleteness and uncertainty of monitoring data, it is necessary to use DST for integrating the data of different monitoring indexes. 

In the multisource data system, there exist tree-level data structures to describe the complexity of data type. Data from a monitoring index belongs to the homologous information. Data between different monitoring items belongs to heterogeneous information. Therefore, it is unsuitable to integrate all information just by DST. For the homologous information, the data provided by different sensors does not have the same degree of reliability. Taking the sensors monitoring the horizontal displacement for example, measurements can differ from one sensor to another in term of reliability. Therefore, the data from homologous information should be modified according to their reliabilities. The dynamic reliability based on the supporting degree is used to modify the homologous information.

As for the heterogeneous information, there exist fuzziness and uncertainty between different monitoring items. We must consider that heterogeneous information is obtained from different monitoring items, and there may even exist conflicts between information from different sources. For example, displacements of a dam are considered in a safe range while uplift pressures are over the safe range. To reflect the fuzziness and uncertainty of heterogeneous information, the interval-valued intuitionistic fuzzy set is employed to describe the variation between heterogeneous information. The fuzzy set is a powerful mathematical framework for handling uncertain, imprecision, and ambiguous information [8]. Fuzzy set has been applied successfully to various areas both in theoretical and applicable aspects such as pattern recognition, data mining, etc. Liu et al. [9] proposed a method based on the sentiment analysis technique and the intuitionistic fuzzy set theory to rank the products through online reviews. Rokni et al. [10] developed a new framework for optimizing shop scheduling using fuzzy set theory. Sooraj [11] proposed a new algorithm by applying the fuzzy soft set theory in group decision making. Dogan et al. [12] proposed a fuzzy decision model combining analytic hierarchy process and technique for order of preference by similarity to ideal solution with intuitionistic fuzzy sets to locate autonomous vehicles. Deveci et al. [13] used interval-valued intuitionistic fuzzy with quality function deployment methodology to evaluate the service quality in public bus transportation. The interval-valued intuitionistic fuzzy set (IVIFS) is another form of fuzzy set. In the interval-valued intuitionistic fuzzy set, the membership degree and non-membership degree are in the form of interval number. 

In this paper, the dam safety assessment model based on interval-valued intuitionistic fuzzy set and evidence theory is proposed to evaluate the safety reliability for dams. In the model, the dynamic reliability based on the supporting degree is used to modify the data from homologous information. The interval-valued intuitionistic fuzzy set is employed to describe the uncertainty and fuzziness between heterogeneous information, and the DST is applied to integrating the data from heterogeneous information. To validate the feasibility of the model, a multiple-arch dam experiencing reinforcement is taken as an example. Through the fusion results of the multiple-arch dam data, the potential risk is identified to be located at the dam section #13 and the dam become safer and more stable after reinforcement.

The rest of the paper is organized as follows: Section 2.1 presents a brief description of the framework of the multisource model and risk assessment system. The interval-valued intuitionistic fuzzy set, the dynamic reliability, and the Dempster-Shafer evidence theory are introduced in Section 2.2 and Section 2.3. Section 2.4 describes the solving process of the dam safety assessment model. The description of a case study is presented in Section 3.1. The fusion process of the dam risk assessment and the comparison among other model are discussed in Section 3.2, Section 3.3 and Section 3.4. The fusion results of the multiple-arch dam before and after reinforcement are presented in Section 4. Finally, the conclusions and research plan for the future work are given in Section 5.

## 2. Construction Principle 

### 2.1. Framework of the Multisource Information Fusion-Based Model

**Figure 1:**
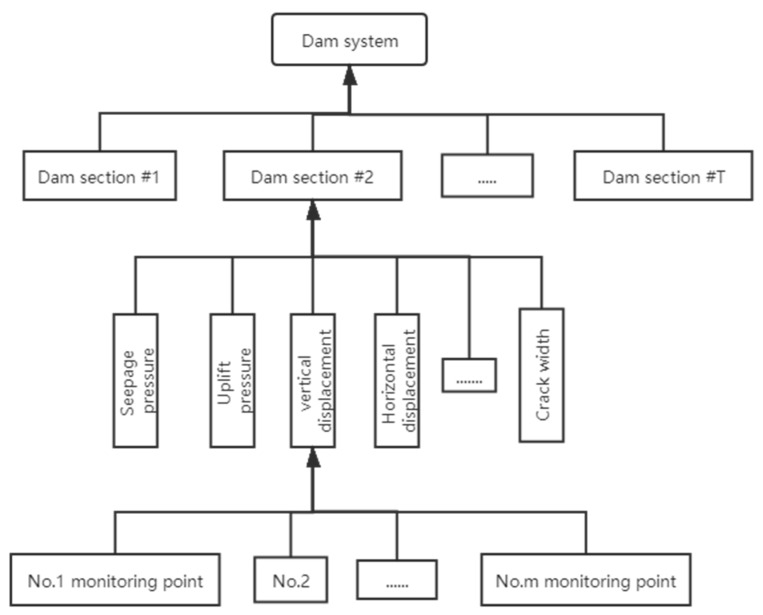
The framework of dam safety assessment.

There are many factors influencing the safety of a dam in the dam safety assessment, including deformation and cracks of the dam body, seepage of the dam foundation, etc. [7]. The dam safety is related to the security of each dam section shown in Figure 1. Every part can serve as a subsystem of the whole system. Every subsystem has several monitoring items (like vertical displacement, seepage pressure, and crack width). There are many monitoring points for a monitoring item. For example, there are several monitoring sensors installed in the dam to monitor vertical displacements. Therefore, a lot of quantity information with certainty and fuzzy information with uncertainty can be collected from dam monitoring activities.

The multisource fusion-based model consists of three level fusion models. The first fusion model is responsible for the fusion of monitoring points, which aims at analyzing the association between the homologous information, extracting the common features, and integrating the homologous information. The second and third fusion models are used for fusion of monitoring items and dam sections, respectively. The information from monitoring items and dam sections is the heterogeneous information. Therefore, two fusion models are used to construct the links between heterogeneous information and obtaining the higher-level results.

### 2.2. Risk Assessment System of Single Monitoring Point

Monitoring points are located at the first-level model shown in Figure 1. For example, there are many monitoring points for the vertical displacements. The measurements vary from one monitoring point to others. Therefore, judgement criteria of dam structure are employed to describe the safety reliability of each monitoring point according to the measurement variation [14]. There are five evaluation indexes in the judgement criteria of dam structure. Five evaluation indexes describing dam behavior can be written as follows:

(1) $$\begin{array}{ll} V & =\left\{V_{1},\cdots ,V_{5}\right\}\\ & =\left\{Normal,\;Nearly\;normal,\;Mildly\;abnormal,\;Severely\;abnormal,\;Malignant\;abnormal\;\right\}\\ K & =\{\left[0.8,\;1\right],\left[0.6,\;0.8\right),\left[0.4,\;0.6\right),\left[0.2,\;0.4\right),[0,\;0.2)\} \end{array}$$

where *K* represents the reliability degrees of these evaluation indexes. Taking *V*_1_ for example, [0.8,1] represents the range of the reliability degree of the first evaluation index.

Five evaluation indexes represent five safety states from trend performance of monitoring data. The dam remains normal if the measurements of monitoring points can be forecasted accurately in trend performance. In the analysis of trend performance, the mathematical relationships between monitoring items and their factors such as water levels, temperatures, and time-varying effects should be determined first [15]. The mathematical relationship of monitoring item can be described as the following equation:

(2) $$y=\sum_{i=1}^{4}a_{i}H^{i}+\sum_{i=1}^{m}b_{i}T_{i}+c_{1}\theta +c_{2}\ln\theta$$

where *y* is the monitoring item (displacement, crack width, uplift pressure, and so on), *H* is the water head difference between upstream and downstream, *T_i_* is the temperature (water temperature, air temperature, concrete temperature, and so on), *m* is the number of temperature variables, *θ* is the time-varying variable, and *a_i_, b_j_, c*_1_, *c*_2_ are the parameters.

**Figure 2:**
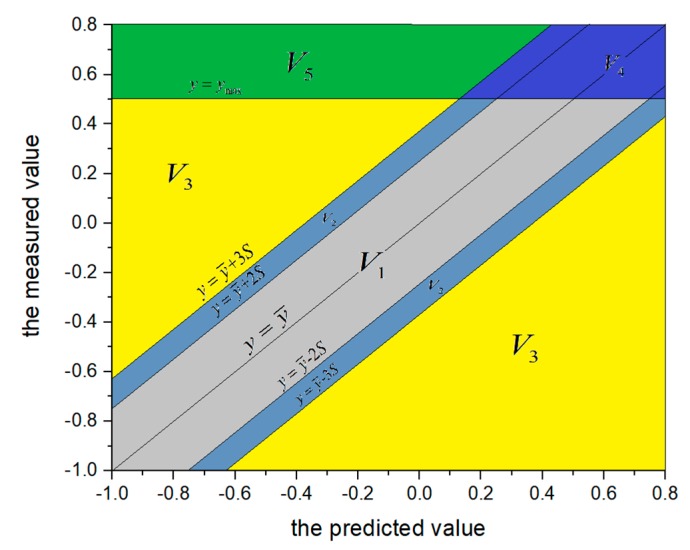
The diagram about risk assessment for the evaluation indexes.

To obtain the parameters in Equation (2), the monitoring data is partitioned into the training samples and test samples. For the training samples, the least square method is used to calculate the parameters of the mathematical equation. The test samples are used to analyze the dam safety state during the time period of test samples [16]. Given the calculated mathematical equation $\bar{y}=G\left(H,T,\theta \right)$, the five evaluation indexes in Figure 2 are defined in Table 1.

Suppose that the number of test samples under the *i*th monitoring point is *T*, the basic probability assignment (BPA) $m_{i}$ of *i*th monitoring point can be written as:

(3) $$m_{i}:m_{i}\left(\left\{V_{j}\right\}\right)=\frac{n_{j}}{T_{i}}k_{j}\left(j=1,2,3,4,5\right),m_{i}\left(\left\{ο\right\}\right)=1-\sum_{j=1}^{5}m_{i}\left(\left\{V_{j}\right\}\right)$$

where *k_j_* is the reliability degree of *j*th evaluation index, *n_j_* is the number of test samples belonging to the *j*th evaluation index, and *m_i_*({o}) represents the unknown degree of the monitoring point.

From the framework of the risk assessment, the BPA of each monitoring point is independent. In some case, there exist some conflicts between assessment results of monitoring points. For example, the dam remains malignant abnormal according to the BPA of one monitoring point while the dam remains normal judged from other monitoring points. The reliability of fusion result will be reduced if these BPAs of monitoring points are integrated directly. Therefore, the BPAs of all monitoring points should be modified before the fusion process.

### 2.3. Modifying the BPAs to the Interval-Valued Intuitionistic Fuzzy Sets

Recently, a modification method based on the dynamic reliability was proposed to revise the BPAs of monitoring points [17]. The dynamic reliability represents the ability of a sensor to provide information accurately, which is influenced by many factors specific to the sensor. For instance, each displacement sensor is different in term of completeness, precision, and certainty. Additionally, the working environment can also affect the dynamic reliability. However, it is difficult and inconvenient to assess the reliability of each sensor through the equipment. From the principle of majority, the dynamic reliability of a sensor increases with the similarity degree between its reading and readings of other sensors. Therefore, the dynamic reliability based on the supporting degree is employed to modify the BPAs. The supporting degree can be considered as a symmetric measurement between BPAs according to the distance and similarity [18]. The calculation of supporting degree is based on the relationship between belief function and intuitionistic fuzzy sets. To explain the relationship, the basic concepts of Dempster-Shafer evidence theory and fuzzy sets are introduced below.

#### 2.3.1. The Basic Probability Assignment in Dempster-Shafer Evidence Theory

Dempster-Shafer theory (DST) is a recursive theory based on decision theory and evidence theory, which can be interpreted as a generalization of probability theory [19]. DST is a powerful tool in handling the fuzzy data fusion and has a good performance in solving fuzzy decision-making problem [20,21]. DST is based on a finite set consisting of mutually exclusive elements, called the frame of discernment. In DST, the frame of discernment contains all the combination of the possible situations. The basic of DST is the basic probability assignment function. The belief and plausibility function are the upper and lower limits of support interval on one assumption [19].

Let Θ = {*θ*_1_, *θ*_2_, …, *θ*_3_} be the frame of discernment. A basic probability assignment (BPA) is a function *m*: 2^Θ^→[0,1], satisfying the following conditions:

(4) $$m\left(\phi \right)=0,\;\sum_{A\subseteq \Theta }m(A)=1$$

where ϕ denotes empty set, and *A* is any subset of Θ. Such a function is also called a belief structure.

The belief function *Bel*(*A*) and plausibility function *Pl*(*A*) are two important functions in the belief structure. *Bel*(*A*) represents all basic probability masses assigned exactly to *A* and its smaller subsets, and *Pl*(*A*) represents all possible basic probability masses that could be assigned to *A* and its smaller subsets. Given a belief structure *m* on Θ, the belief function and plausibility function can be defined respectively as follows:

(5) $$Bel\left(A\right)=\sum_{B\subseteq A}m\left(B\right),\;Pl\left(A\right)=\sum_{B\cap A\neq \phi }m\left(B\right)=1-\sum_{B\cap A=\phi }m\left(B\right)$$

where *Bel*(*A*) and *Pl*(*A*) can be interpreted as the lower and upper bounds of probability to which *A* is supported.

Considering that the Dempster’s combination rule is employed to integrate the heterogeneous information in Section 2.4, the basic concepts of the rule are introduced in this section.

Given two belief structures *m*_1_ and *m*_2_ on Θ, the combined belief structure *m*_12_ from the application of Dempster’s combination rule can be obtained as follows:

(6) $$m_{12}\left(A\right)=\{\begin{array}{c} \frac{\sum_{B\cap C=A}m_{1}\left(B\right)m_{2}\left(C\right)}{1-\sum_{B\cap C=\phi }m_{1}\left(B\right)m_{2}\left(C\right)},\forall A\subseteq \Theta ,A\neq \phi \\ 0,\;A=\phi \end{array}$$

When multiple independent sources of evidence are available, the combined evidence can be obtained as follows:

(7) $$m\left(A\right)=\{\begin{array}{c} \frac{\sum_{\cap A_{j}=A}\prod_{i=1}^{n}m_{i}\left(A_{j}\right)}{1-\sum_{\cap A_{j}=\phi }\prod_{i=1}^{n}m_{i}\left(A_{i}\right)},\forall A\subseteq \Theta ,\;A\neq \phi \\ 0,A=\phi \end{array}$$

where *n* is the number of evidence pieces, *i* denotes the *i*-th piece of evidence, *m_i_*(*A_j_*) is the BPA of hypothesis *A_j_* supported by evidence *i*, and *m*(*A*) reflects the combined belief structure from *n* mutually independent sources of evidence.

From the basic concepts of DST, the BPA obtained from the risk assessment system can serve as a piece of evidence under the frame of discernment *V* = {*V*_1_, *V*_2_, …, *V*_5_}. The fusion process of monitoring points can be served as the combination of multiple independent sources of evidence.

#### 2.3.2. Considering the Basic Probability Assignment in the View of the Intuitionistic Fuzzy Set

The fuzzy set (FS) concept proposed by Zadeh is the most successful mathematical framework for handling uncertain, imprecise and ambiguous information [22]. Fuzzy sets have been applied successfully to various areas both in theoretical and applicable aspects such as pattern recognition, data mining, economics, artificial intelligence, decision making problems, etc. [23,24]. In the decision-making process, uncertainties play a dominant role and it is very difficult to get an accurate decision without handling them. To solve these problems, the fuzzy set (FS) is proposed to present the information with a membership that lies between zero and one. However, there usually exist neutrals during the decision-making process in real-life situation. Thus, the hesitation degree is introduced to present the information about these neutrals. Compared with the traditional fuzzy set, the intuitionistic fuzzy set (IFS) consists of acceptance degree, rejection degree and hesitation degree [25].

Firstly, let us consider the basic concepts of FS, IFS, IVIFS.

**Definition** **1.** *Fuzzy set: A FS A in X = {x*_1_*, x_*2*_, …, x_n_} is characterized by a membership degree μ_A_(x_i_) with a range in* [0.1]. *The fuzzy set can be represented in the following way:*

(8) $$A=\left\{\langle x_{i},\mu_{A}\left(x_{i}\right)\rangle |x_{i}\in X\right\}$$

**Definition** **2.** *Intuitionistic fuzzy set: An IFS A in X = {x_*1*_, x_*2*_, …, x_n_} is characterized by a membership degree μ_A_(x_i_) and a non-membership degree ν_A_(x_i_) with a range in* [0,1]*. The intuitionistic fuzzy set can be represented as a triplet in the following way:*

(9) $$A=\left\{\langle x_{i},\mu_{A}\left(x_{i}\right),\nu_{A}\left(x_{i}\right)\rangle |x_{i}\in X\right\}$$

*where μ_A_(x_i_), ν_A_(x_i_) should satisfy the following condition μ_A_(x_i_) + ν_A_(x_i_) ≤ *1*. For each A ∈ IFS(X), the hesitation degree π_A_(x_i_) can be obtained from the condition π_A_(x_i_) = *1* − μ_A_(x_i_) − ν_A_(x_i_*).

**Definition** **3.** *Intersection between IFSs: For two IFSs A, B in X = {x_*1*_ , x_*2*_ , …, x_n_}, the intersection between A and B can be defined as follows:*

(10) $$C=A\cap B=\left\{\langle x,\min\left(\mu_{A}\left(x\right),\mu_{B}\left(x\right)\right),\max\left(\nu_{A}\left(x\right),\nu_{B}\left(x\right)\right)\rangle |x\in X\right\}$$

*where A* = {〈*x_i_,μ_A_(x_i_),ν_A_(x_i_)*〉|*x_i_∈X*} *and B =* {〈*x_i_,μ_B_(x_i_),ν_B_(x_i_)*〉|*x_i_∈X*}.

**Definition** **4.** *Distance between IFSs: For two IFSs A, B in X = {x_*1*_, x_*2*_, …, x_n_}, the normalized Euclidean distance between A and B can be defined as follows:*

(11) $$D_{E}\left(A,B\right)=\sqrt{\frac{1}{2n}\sum_{i=1}^{n}\left({\left(\mu_{A}\left(x_{i}\right)-\mu_{B}\left(x_{i}\right)\right)}^{2}+{\left(\nu_{A}\left(x_{i}\right)-\nu_{B}\left(x_{i}\right)\right)}^{2}+{\left(\pi_{A}\left(x_{i}\right)-\pi_{B}\left(x_{i}\right)\right)}^{2}\right)}$$

*where A = *{〈*x_i_,μ_A_(x_i_),ν_A_(x_i_)*〉*|x_i_∈X} and B = {〈x_i_,μ_B_(x_i_),ν_B_(x_i_)〉|x_i_∈X*}.

**Definition** **5.** *Interval-valued intuitionistic fuzzy set: An IVIFS A in X = {x_*1*_, x_*2*_, …, x_n_} is characterized by an interval of membership degree* [$\mu_{A}^{l}\left(x_{i}\right),\mu_{A}^{u}(x_{i}$)] *and an interval of non-membership degree* [$\nu_{A}^{l}\left(x_{i}\right),\nu_{A}^{u}(x_{i}$)]. *Interval-valued intuitionistic fuzzy set can be represented in the following way:*

(12) $$A=\left\{\langle x_{i},\left[\mu_{A}^{l}\left(x_{i}\right),\mu_{A}^{u}\left(x_{i}\right)\right],\left[\nu_{A}^{l}\left(x_{i}\right),\nu_{A}^{u}\left(x_{i}\right)\right]\rangle |x_{i}\in X\right\}$$

*where $\mu_{A}^{l}\left(x_{i}\right),\;\mu_{A}^{u}(x_{i}$) are the lower and upper bound of the membership degree, respectively, $\nu_{A}^{l}\left(x_{i}\right),\;\nu_{A}^{u}(x_{i}$) are the lower and upper bound of the non-membership degree respectively. $\mu_{A}^{l}\left(x_{i}\right),\;\mu_{A}^{u}(x_{i}$), $\nu_{A}^{l}\left(x_{i}\right)$ and $\;\nu_{A}^{u}\left(x_{i}\right)$ satisfy the following condition: $\mu_{A}^{u}\left(x_{i}\right)+\nu_{A}^{u}\left(x_{i}\right)\leq 1$ with a range in *[0,1]*. $\pi_{A}^{l}\left(x_{i}\right),\;\pi_{A}^{u}(x_{i}$) are the lower and upper bound of the hesitation degree, respectively. $\pi_{A}^{l}\left(x_{i}\right),\;\pi_{A}^{u}(x_{i}$) satisfy the following conditions: $\pi_{A}^{l}\left(x_{i}\right)=1-\mu_{A}^{u}\left(x_{i}\right)-\nu_{A}^{u}\left(x_{i}\right)$ and $\pi_{A}^{u}\left(x_{i}\right)=1-\mu_{A}^{l}\left(x_{i}\right)-\nu_{A}^{l}\left(x_{i}\right).\;$ If $\mu_{A}^{l}\left(x_{i}\right)=\;\mu_{A}^{u}(x_{i}$) and $\nu_{A}^{l}\left(x_{i}\right)=\nu_{A}^{u}(x_{i}$), A will become the IFS.*

**Definition** **6.** *Distance between IVIFSs: For two IVIFSs A, B in X = {x*_1_ *, x*_2_ *, …, x_n_}, the distance between A and B can be defined as follows:*

(13) $$D\left(A,B\right)=\frac{1}{4n}\sum_{i=1}^{n}\left[\left|\mu_{A}^{l}\left(x_{i}\right)-\mu_{B}^{l}\left(x_{i}\right)\right|+\left|\mu_{A}^{u}\left(x_{i}\right)-\mu_{B}^{u}\left(x_{i}\right)\right|+\left|\nu_{A}^{l}\left(x_{i}\right)-\nu_{B}^{l}\left(x_{i}\right)\right|+\left|\nu_{A}^{u}\left(x_{i}\right)-\nu_{B}^{u}\left(x_{i}\right)\right|+\left|\pi_{A}^{l}\left(x_{i}\right)-\pi_{B}^{l}\left(x_{i}\right)\right|+\left|\pi_{A}^{u}\left(x_{i}\right)+\pi_{B}^{u}\left(x_{i}\right)\right|\right]$$

*where A = $\left\{\langle x_{i},\;\left[\mu_{A}^{l}\left(x_{i}\right),\;\mu_{A}^{u}\left(x_{i}\right)\right],\left[\nu_{A}^{l}\left(x_{i}\right),\;\nu_{A}^{u}\left(x_{i}\right)]\rangle \right|x_{i}\in X\right\}$ and B = $\left\{\langle x_{i},\;\left[\mu_{B}^{l}\left(x_{i}\right),\;\mu_{B}^{u}\left(x_{i}\right)\right],\;\left[\nu_{B}^{l}\left(x_{i}\right),\;\nu_{B}^{u}\left(x_{i}\right)]\rangle \right|x_{i}\in X\right\}$.*

From the basic concepts of IFS, an IFS 〈*μ_A_*(*x*), *_A_*(*x*)〉 has some physical interpretations. Taking 〈*μ_A_*(*x*), *_A_*(*x*)〉 = 〈0.2,0.3〉 for example, the IFS can be interpreted as a voting model where the votes are two in favor, three against, and five abstentions. From the basic concepts of DST, the belief and plausibility functions can be interpreted as the lower and upper bounds of probability in the framework of evidence theory. If the BPA is considered as an IFS, the belief function represents the two men who absolutely vote in favor and the plausibility function represents the seven men who could vote in favor. From the transformation from a BPA *m* to an IFS *A* in Θ = {*θ*_1_, *θ*_2_, …, *θ_n_*}, *Bel*(*θ_i_*) and 1 − *Pl*(*θ_i_*) can serve as the membership degree *μ_A_*(*x_i_*) and the non-membership degree *ν_A_*(*x_i_*). Based on such an analysis, a BPA *m* on the discernment Θ = {*θ*_1_, *θ*_2_, …, *θ_n_*} can be transformed to an IFS *A* in Θ = {*θ*_1_, *θ*_2_, …, *θ_n_*}. The corresponding IFS can be expressed as follows:

(14) $$A=\left\{\langle \theta_{i},\mu_{A}\left(\theta_{i}\right),\nu_{A}\left(\theta_{i}\right)\rangle |\theta_{i}\in \Theta \right\}=\left\{\langle \theta_{i},Bel\left(\theta_{i}\right),1-Pl\left(\theta_{i}\right)\rangle |\theta_{i}\in \Theta \right\}$$

From the analysis above, each BPA derived from the risk assessment system of monitoring points can be transformed into an IFS defined in *V* = {*V*_1_, *V*_2_, …, *V*_5_}. The BPA *m_j_* on the discernment frame *V* = {*V*_1_,*V*_2_,…,*V*_5_} can be transformed to the IFS *A_j_* = {〈*V_i_,μ*(*V_i_*),*ν*(*V_i_*)〉|*V_i_*∈*V*} as follows:

(15) $$\mu \left(V_{i}\right)=m_{j}\left(\left\{V_{i}\right\}\right),\;\nu \left(V_{i}\right)=1-\left(m_{j}\left(\left\{V_{i}\right\}\right)+m_{j}\left(\left\{ο\right\}\right)\right)=\sum_{\begin{array}{l} k=1\\ k\neq i \end{array}}^{5}m_{j}\left(\left\{V_{k}\right\}\right)$$

where *m_j_*({*V_i_*}) is the membership degree belonging to the *i*-th evaluation index for the *j*-th monitoring point, *m_j_*({*o*}) is the unknown degree for the *j*-th monitoring point.

From the basic concepts of DST and IFS, it is feasible to transform BPAs and IFSs in *V* = {*V*_1_, *V*_2_, …, *V*_5_}. Then the supporting degrees can be calculated according to the distances among IFSs of monitoring points.

#### 2.3.3. Extracting the IVIFSs According to the Dynamic Reliability of Monitoring Points

The concept of supporting degree between IFSs has been proposed in some modified evidence combination rules [26,27]. The supporting degree can be considered as a symmetric measurement between IFSs according to the similarity degree. The similarity degree between IFSs is related with the intersection of them. Recently, many methods have been proposed to define the similarity measures for IFSs. The similarity for IFSs based on Euclidean distance is served as the supporting degree between IFSs [17]. Let *A*_1_ and *A*_2_ be two IFSs in *X* = {*x*_1_, *x*_2_, …, *x_n_*}, the supporting degree *Sup*(*A*_1_, *A*_2_) can be described as follows:

(16) $$\begin{array}{ll} Sup\left(A_{1},A_{2}\right) & =Sim\left(A_{1},A_{1}\cap A_{2}\right)=1-Dis\left(A_{1},A_{1}\cap A_{2}\right)\\ & {=1-D}_{E}\left(A_{1},A_{1}\cap A_{2}\right)=1-\sqrt{\frac{1}{2n}\sum_{i=1}^{n}\left({\left(\mu_{A_{1}}\left(x_{i}\right)-\mu_{C}\left(x_{i}\right)\right)}^{2}+{\left(\nu_{A_{1}}\left(x_{i}\right)-\nu_{C}\left(x_{i}\right)\right)}^{2}+{\left(\pi_{A_{1}}\left(x_{i}\right)-\pi_{C}\left(x_{i}\right)\right)}^{2}\right)} \end{array}$$

where $C=\left\{\langle x,\mu_{C}\left(x_{i}\right),\nu_{C}\left(x_{i}\right)\rangle |x_{i}\in X\right\}$ is the intersection between *A*_1_ and *A*_2_.

As mentioned above, the dynamic reliability of a sensor increases with the similarity degree between its reading and other readings. The greater the amount of supporting degrees between the monitoring point and others is, the higher the dynamic reliability of the monitoring point is.

Suppose that the number of monitoring points is *N* and the BPA provided by each monitoring point is *m_N_*, the supporting degree matrix (SDM) is expressed as [18]:

(17) $$SDM=\left(\begin{array}{cccc} Sup\left(m_{1},m_{1}\right) & Sup\left(m_{1},m_{2}\right) & \cdots & Sup\left(m_{1},m_{N}\right)\\ Sup\left(m_{2},m_{1}\right) & Sup\left(m_{2},m_{2}\right) & \cdots & Sup\left(m_{2},m_{N}\right)\\ \vdots & \vdots & \ddots & \vdots \\ Sup\left(m_{N},m_{1}\right) & Sup\left(m_{N},m_{2}\right) & \cdots & Sup\left(m_{N},m_{N}\right) \end{array}\right)$$

The dynamic reliability of each monitoring point can be defined as the relative total supporting degrees of BPAs. The dynamic reliability can be described as:

(18) $$Total\_Sup\left(m_{i}\right)=\sum_{\begin{array}{l} j=1\\ j\neq i \end{array}}^{N}Sup\left(m_{j},m_{i}\right)$$

(19) $$R\left(m_{i}\right)=\frac{Total\_Sup\left(m_{i}\right)}{\underset{j=1,2,\cdots ,N}{\max}\left\{Total\_Sup\left(m_{j}\right)\right\}}$$

where *R*(*m_i_*) is the dynamic reliability of the *i*th monitoring point, *Total_Sup*(*m_i_*) is the total supporting degree of *m_i_* obtained from all other BPAs.

For all monitoring points, the monitoring point with the highest relative reliability is regarded as the primary monitoring point and the BPAs of other monitoring points can be updated according to the absolute dynamic reliability of each monitoring point as follows:

(20) $$m_{i}^{R}\left(\left\{\theta_{k}\right\}\right)=m_{i}\left(\left\{\theta_{k}\right\}\right)R\left(m_{i}\right)\;m_{i}^{R}\left(ο\right)=1-\sum_{k=1}^{K}m_{i}^{R}\left(\left\{\theta_{k}\right\}\right)$$

where $m_{i}^{R}\left(\left\{\theta_{k}\right\}\right)$ is the BPA of the *i*-th monitoring point after modification.

Then the intuitionistic fuzzy set $A_{j}^{\prime }=\left\{\langle V_{i},\mu^{\prime }\left(V_{i}\right),\pi^{\prime }\left(V_{i}\right)\rangle |V_{i}\in V\right\}$ can be described as follows:

(21) $$\mu^{\prime }\left(V_{i}\right)=m_{j}^{R}\left(\left\{V_{i}\right\}\right),\;\nu^{\prime }\left(V_{i}\right)=1-\left(m_{j}^{R}\left(\left\{V_{i}\right\}\right)+m_{j}^{R}\left(\left\{ο\right\}\right)\right)=\sum_{\begin{array}{l} k=1\\ k\neq i \end{array}}^{5}m_{j}^{R}\left(\left\{V_{k}\right\}\right)$$

where $A_{j}^{\prime }$ is the intuitionistic fuzzy set of the *j*-th monitoring point after modification.

After modifying the original BPAs from all the monitoring points, the interval-valued intuitionistic fuzzy set is employed to describe the safety degree of these monitoring points. The IVIFS $M=\left\{\langle V_{i}\left[\mu_{A}^{l}\left(V_{i}\right),\mu_{A}^{u}\left(V_{i}\right)\right],\left[\nu_{A}^{l}\left(V_{i}\right),\nu_{A}^{u}\left(V_{i}\right)]\rangle \right|V_{i}\in V\right\}$ can be expressed as follows:

(22) $$\left\{ \begin{array}{l} \mu_{A}^{l}\left(V_{i}\right)=\underset{j=1,2,\cdots ,N}{\max}\left\{\mu_{j}^{\prime }\left(V_{i}\right)\right\}\;\mu_{A}^{u}\left(V_{i}\right)=\underset{j=1,2,\cdots ,N}{\min}\left\{\mu_{j}^{\prime }\left(V_{i}\right)\right\}\\ \nu_{A}^{l}\left(V_{i}\right)=\underset{j=1,2,\cdots ,N}{\max}\left\{\nu_{j}^{\prime }\left(V_{i}\right)\right\}\;\nu_{A}^{u}\left(V_{i}\right)=\underset{j=1,2,\cdots ,N}{\min}\left\{\nu_{j}^{\prime }\left(V_{i}\right)\right\} \end{array}\right.$$

where *M* is the IVIFS of the monitoring item and the intuitionistic fuzzy set of the *j*-th monitoring point is $A_{j}^{\prime }=\left\{\langle V_{i},\mu^{\prime },\left(V_{i}\right),\nu_{j}^{\prime }\left(V_{i})\rangle \right|V_{i}\in V\right\}$.

### 2.4. The Fusion Process Based on IVIFS and DST

In the framework of the dam safety assessment, the BPAs of monitoring points are transformed to IVIFS of monitoring items in the first fusion model. Then the IVIFSs of monitoring items are merged into IVIFS of the dam sections in the second fusion model. Finally, IVIFSs of dam sections are merged into the IVIFS of the dam in the third fusion model. Considering the randomness, uncertainty and fuzziness between heterogeneous information, the Dempster’s combination rule is used to integrate IVIFSs in the second and third fusion models. The arithmetic rules for IVIFSs can be described as follows:

Suppose that the dam sections are *E* = {*E*_1_,*E*_2_,…,*E_T_*}, the monitoring items are *A* = {*A*_1_,*A*_2_,…,*A_n_*} and the evaluation indexes are *V* = {*V*_1_,*V*_2_,…,*V*_5_}, the interval-valued intuitionistic fussy set of a certain dam section *E_k_* can be expressed as follows:

(23) $$IVIFSs\;E_{k}=\left[\begin{array}{ccc} \left\{V_{1},\langle \mu_{A_{1},k}^{l}\left(V_{1}\right),\mu_{A_{1},k}^{u}\left(V_{1}\right)\rangle ,\langle \nu_{A_{1},k}^{l}\left(V_{1}\right),\nu_{A_{1},k}^{u}\left(V_{1}\right)\rangle \right\} & \cdots & \left\{V_{1},\langle \mu_{A_{n},k}^{l}\left(V_{1}\right),\mu_{A_{n},k}^{u}\left(V_{1}\right)\rangle ,\langle \nu_{A_{n},k}^{l}\left(V_{1}\right),\nu_{A_{n},k}^{u}\left(V_{1}\right)\rangle \right\}\\ \vdots & \cdots & \vdots \\ \left\{V_{5},\langle \mu_{A_{1},k}^{l}\left(V_{5}\right),\mu_{A_{1},k}^{l}\left(V_{5}\right)\rangle ,\langle \nu_{A_{1},k}^{l}\left(V_{5}\right),\nu_{A_{1},k}^{l}\left(V_{5}\right)\rangle \right\} & \cdots & \left\{V_{5},\langle \mu_{A_{n},k}^{l}\left(V_{5}\right),\mu_{A_{n},k}^{u}\left(V_{5}\right)\rangle ,\langle \nu_{A_{n},k}^{l}\left(V_{5}\right),\nu_{A_{n},k}^{l}\left(V_{5}\right)\rangle \right\} \end{array}\right]$$

where $\left\{V_{j},\langle \mu_{A_{i},k}^{l}\left(V_{j}\right),\mu_{A_{i},k}^{u}\left(V_{j}\right)\rangle ,\langle \nu_{A_{i},k}^{l}\left(V_{j}\right),\nu_{A_{i},k}^{u}\;\left(V_{j}\right)\rangle |V_{j}\in V\right\}$ represents the IVIFS of the *i*-th monitoring item in the *k*-th dam section.

In the process of dam safety assessment, there are differences between dam sections and monitoring items. Taking the stress and displacement sensors for example, these sensors are embedded in the heel, toe and many other locations of a dam. Because of their different locations and importance, different weights should be given to the dam sections and monitoring items in the fusion process. To obtain the evaluation weights objectively, the intuitionistic fuzzy entropy is employed to identify the suitable evaluation weights.

**Definition** **7.** *Intuitionistic fuzzy entropy: Suppose that *$C_{l}^{k}=\left\{V_{j},\langle \mu_{A_{i},k}^{l}\left(V_{j}\right),\mu_{A_{i},k}^{u}\left(V_{j}\right)\rangle ,\langle \nu_{A_{i},k}^{l}\left(V_{j}\right),\nu_{A_{i},k}^{u}\;\left(V_{j}\right)\rangle |V_{j}\in V\right\}$*, the intuitionistic fuzzy entropy *$E(C_{i}^{k}$) *can be expressed as follows* [28]:

(24) $$E\left(C_{i}^{k}\right)=\frac{\min\left\{D\left(C_{i}^{k},P\right),D\left(C_{i}^{k},Q\right)\right\}}{\max\left\{D\left(C_{i}^{k},P\right),D\left(C_{i}^{k},Q\right)\right\}}$$

*where P and Q represent the absolutely positive and negative IVIFSs, respectively.* *P* = {*V_j_*,〈1,1〉,〈0,0〉|*V_j_* ∈ *V*}, *Q* = {*V_j_*,〈0,0〉,〈1,1〉|*V_j_* ∈ *V*}.

According to the intuitionistic fuzzy entropy obtained above, the comprehensive weight of *i*th monitoring item can be expressed as follows:

(25) $$\omega_{i}^{k}=\frac{1-E\left(C_{i}^{k}\right)}{n-\sum_{i=1}^{n}E\left(C_{i}^{k}\right)},i=1,2,\cdots ,n$$

The comprehensive weight of the dam section *E_k_* can be expressed as follows:

(26) $$\lambda_{k}=\frac{1-M_{k}}{t-\sum_{k=1}^{t}M_{k}},k=1,2,\cdots ,T$$

where *M_k_* is the average value of the evaluation matrix *E_k_*, $M_{k}=\sum_{i=1}^{n}E\left(C_{i}^{k}\right)/n$.

**Figure 3:**
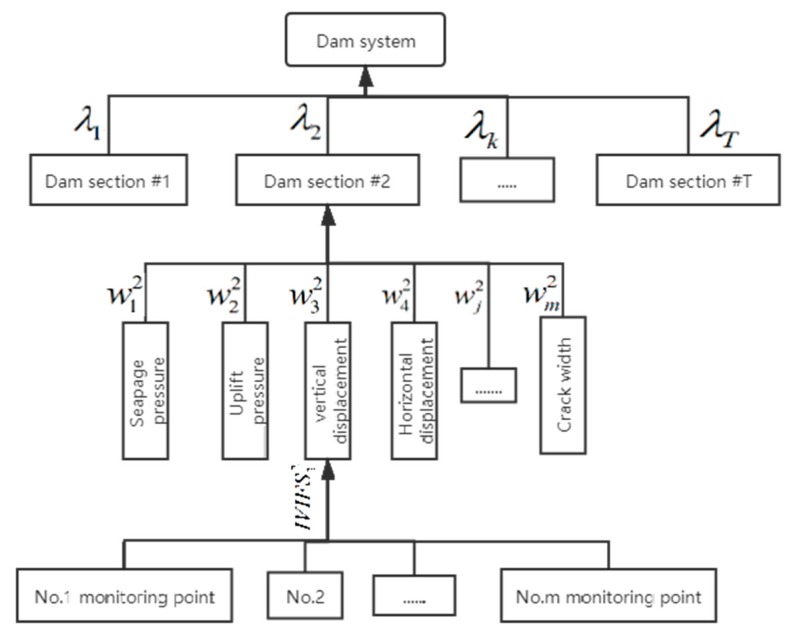
The framework of dam safety assessment after obtaining the weights.

After calculating the weights, the framework of the dam risk assessment model is updated as shown in Figure 3. A new IIVFS information aggregation operation based on evidence theory is used to integrate the IVIFSs. In this section, the fusion process of the *k*-th dam section is employed to describe the calculation process of the operator.

In order to describe the calculation formulas easily, the IVIFS for the *i*-th monitoring item in the *k*-th dam section $C_{l}^{k}$ can be described as follows:

(27) $$S(C_{i}^{k}(V_{j}))=\{V_{j},[\beta_{r,il}^{k}\left(V_{j}\right),\beta_{r,iu}^{k}\left(V_{j})]\right\}$$

(28) $$\left[\beta_{1,il}^{k}\left(V_{j}\right),\beta_{1,iu}^{k}\left(V_{j}\right)\right]=\left[\mu_{A_{i},k}^{l}\left(V_{j}\right),\mu_{A_{i},k}^{l}\left(V_{j}\right)\right]$$

(29) $$\left[\beta_{2,il}^{k}\left(V_{j}\right),\beta_{2,iu}^{k}\left(V_{j}\right)\right]=\left[\nu_{A_{i},k}^{l}\left(V_{j}\right),\nu_{A_{i},k}^{u}\left(V_{j}\right)\right]$$

Considering the weight of each monitoring item, the probability assignment function $\left[m_{r,il}^{k}\left(V_{j}\right),m_{r,iu}^{k}\left(V_{j}\right)\right]$ and residual probability assignment function $\left[m_{H,il}^{k}\left(V_{j}\right),m_{H,iu}^{k}\left(V_{j}\right)\right]$ can be calculated as follows:

(30) $$\left[m_{r,il}^{k}\left(V_{j}\right),m_{r,iu}^{k}\left(V_{j}\right)\right]=w_{i}^{k}\cdot \left[\beta_{r,il}^{k}\left(V_{j}\right),\beta_{r,iu}^{k}\left(V_{j}\right)\right]$$

(31) $$\left[m_{H,il}^{k}\left(V_{j}\right),m_{H,iu}^{k}\left(V_{j}\right)\right]=\left[1-\sum_{r=1}^{2}m_{r,iu}^{k}\left(V_{j}\right),1-\sum_{r=1}^{2}m_{r,il}^{k}\left(V_{j}\right)\right]$$

where the probability assignment function represents the supporting or disagreeing degree of the *j*th evaluation index after assigning the weights of monitoring items, and the residual probability assignment function represents the unknown degree respectively.

After assigning the weights of these monitoring items, the combinatorial probability assignment function $\left[n_{r,il}^{k}\left(V_{j}\right),mn_{r,iu}^{k}\left(V_{j}\right)\right]$ and residual combinatorial probability assignment function $\left[n_{H,il}^{k}\left(V_{j}\right),n_{H,iu}^{k}\left(V_{j}\right)\right]$ can be obtained as follows:

(32) $$n_{r,il}^{k}\left(V_{j}\right)=\frac{1}{\psi_{1}}\cdot \left(n_{r,(i-1)l}^{k}\left(V_{j}\right)m_{r,il}^{k}\left(V_{j}\right)+n_{r,il}^{k}\left(V_{j}\right)n_{H,(i-1)L}^{k}\left(V_{j}\right)+n_{H,il}^{k}\left(V_{j}\right)m_{r,il}^{k}\left(V_{j}\right)\right)$$

(33) $$n_{r,iu}^{k}\left(V_{j}\right)=\frac{1}{\psi_{2}}\cdot \left(n_{r,(i-1)u}^{k}\left(V_{j}\right)m_{r,iu}^{k}\left(V_{j}\right)+n_{r,iu}^{k}\left(V_{j}\right)n_{H,(i-1)u}^{k}\left(V_{j}\right)+n_{H,iu}^{k}\left(V_{j}\right)m_{r,iu}^{k}\left(V_{j}\right)\right)$$

(34) $$n_{H,il}^{k}\left(V_{j}\right)=\frac{1}{\psi_{1}}\cdot \left(n_{H,(i-1)l}^{k}\left(V_{j}\right)m_{H,il}^{k}\left(V_{j}\right)\right)$$

(35) $$n_{H,iu}^{k}\left(V_{j}\right)=\frac{1}{\psi_{2}}\cdot \left(n_{H,(i-1)u}^{k}\left(V_{j}\right)m_{H,iu}^{k}\left(V_{j}\right)\right)$$

where $\psi_{1}=1-\sum_{q=1}^{2}\sum_{s=1}^{2}n_{q,(i-1)l}^{k}\left(V_{j}\right)m_{s,il}^{k}\left(V_{j}\right)$,$\psi_{2}=1-\sum_{q=1}^{2}\sum_{s=1}^{2}n_{q,(i-1)u}^{k}\left(V_{j}\right)m_{s,iu}^{k}\left(V_{j}\right)$. The fusion process can be used as a fusion of two IVIFSs. The combinatorial probability assignment function $\left[n_{r,il}^{k}\left(V_{j}\right),n_{r,iu}^{k}\left(V_{j}\right)\right]$ can be used as the combined result of all the first to *(j −* 1)-th monitoring items.

Finally, the combined IVIFS of the *k*-th dam section *P^k^* can be obtained:

(36) $$\left[\beta_{rl}^{k}\left(V_{j}\right),\beta_{ru}^{k}\left(V_{j}\right)\right]=\left[\frac{n_{r,nl}^{k}\left(V_{j}\right)-\beta_{Hl}^{k}\left(V_{j}\right)n_{r,nl}^{k}\left(V_{j}\right)}{1-n_{H,nl}^{k}\left(V_{j}\right)},\frac{n_{r,nu}^{k}\left(V_{j}\right)-\beta_{Hl}^{k}\left(V_{j}\right)n_{r,nu}^{k}\left(V_{j}\right)}{1-n_{H,nu}^{k}\left(V_{j}\right)}\right]$$

(37) $$\left[\beta_{Hl}^{k}\left(V_{j}\right),\beta_{Hu}^{k}\left(V_{j}\right)\right]=\left[\sum_{i=1}^{n}w_{j}^{k}\left(1-\sum_{r=1}^{2}\beta_{r,iu}^{k}\left(V_{j}\right)\right),\sum_{i=1}^{n}w_{j}^{k}\left(1-\sum_{r=1}^{2}\beta_{r,il}^{k}\left(V_{j}\right)\right)\right]$$

(38) $$P^{k}=\left(V_{j},\left[\beta_{1l}^{k}\left(V_{j}\right),\beta_{1u}^{k}\left(V_{j}\right)\right],\left[\beta_{2l}^{k}\left(V_{j}\right),\beta_{2u}^{k}\left(V_{j}\right)\right]|V_{j}\in V\right)$$

where the decision matrix *P^k^* is the IVIFS of the *k*-th dam section after the second fusion.

From the calculation process in second fusion model, the fusion operation between IVIFSs of monitoring items in *k*th dam section can be achieved by intuitionistic fuzzy entropy and the Dempster’s combination rule. Considering that the fusion process of second fusion model is similar to the third fusion model, the same fusion operation can be employed in the third fusion model. Finally, the IVIFS of the dam can be obtained in the third fusion model.

### 2.5. The Dam Risk Assessment Model Based on IVIFS and DST

**Figure 4:**
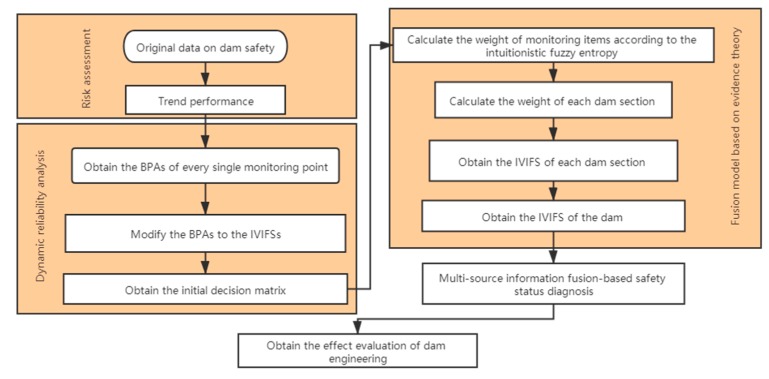
Structure of the proposed dam safety assessment model.

There are many factors that influence the safety of a dam in the dam safety assessment, such as deformation, seepage, crack, etc. There are many monitoring points just for a monitoring item. Therefore, a large of quantity information with certainty and fuzzy information with uncertainty can be collected from dam monitoring activities. The interval-valued intuitionistic fuzzy set is employed to extract the common features and reflect the uncertain variation. The dynamic reliability based on the supporting degree is used to modify the initial evaluation results. An IVIFS information aggregation operation is employed to integrate the heterogeneous information of the second and third fusion model. The structure of the dam risk assessment is showed in Figure 4. Main steps are as follows:

*Step 1*:  Obtain the basic probability assignment of each monitoring point according to the trend performance.
*Step 2*:  Transform the basic probability assignment to the intuitionistic fuzzy set, obtain the supporting degree of each monitoring point, and modify the intuitionistic fuzzy sets.
*Step 3*:  Fuse the intuitionistic fuzzy sets and obtain the initial decision matrix.
*Step 4*:  Calculate the weights of monitoring items and dam sections according to the intuitionistic fuzzy entropy.
*Step 5*:  Obtain the IVIFSs of dam sections according to the IIVFS information aggregation operator.
*Step 6*:  Obtain the IVIFS of the dam.

As showed in Figure 4, dynamic reliability analysis is employed to assess the reliability of each monitoring sensor. There are many sensors embedded in a dam. Taking the stress sensors for example, different sensors are embedded in the heel, toe and many other locations of a dam. Because of their different locations and importance, it is inappropriate to integrate the information directly. According to the dam behavior in normal situation, the monitoring data from these sensors at each dam section shows similar trends. Therefore, the dynamic reliability analysis according to the similarity degree is suitable. Considering that the distribution of these sensors is uneven and there are only a few sensors in certain dam sections, there exist uncertainty and fuzziness between each dam section. Taking a gravity dam for example, there are more stress sensors embedded in the overflow section than displacement sensors. The risk assessment about displacement in overflow section is fuzzier and more uncertain. Therefore, the intuitionistic fuzzy set is used to solve the problem. The exists difference between different monitoring points. Taking the monitoring points of the horizontal displacement for example, the IFSs of several monitoring points indicate that the dam remains normal. The IFSs of other monitoring points indicates that the dam remains severely abnormal. If these IFSs are integrated directly, the fusion result is more likely to ignore the possibility that the dam remains several abnormal. To describe the safety variety of the monitoring points, the interval-valued intuitionistic fuzzy set is employed. A new IVIFS information aggregation operation based on DST is employed to integrate these IVIFSs. Considering the fuzziness and uncertainty between different monitoring items, the intuitionistic fuzzy entropy is used to obtain the weights of monitoring items. A large entropy represents that the risk assessment for the monitoring item is fuzzier and more uncertain. Therefore, it is suitable to assign different weights to monitoring items according to the intuitionistic fuzzy entropy. Considering that the fusion of IVIFSs is heterogeneous fusion, Dempster’s combination rule is used in the second and third fusion models. Then an aggregation operation based on DST is proposed to integrate the IVIFSs in the second and third fusion models. In conclusion, the risk assessment model takes the reliability difference between monitoring sensors, fuzziness among monitoring items, and uncertainty between dam sections into account at the same time.

**Figure 5:**
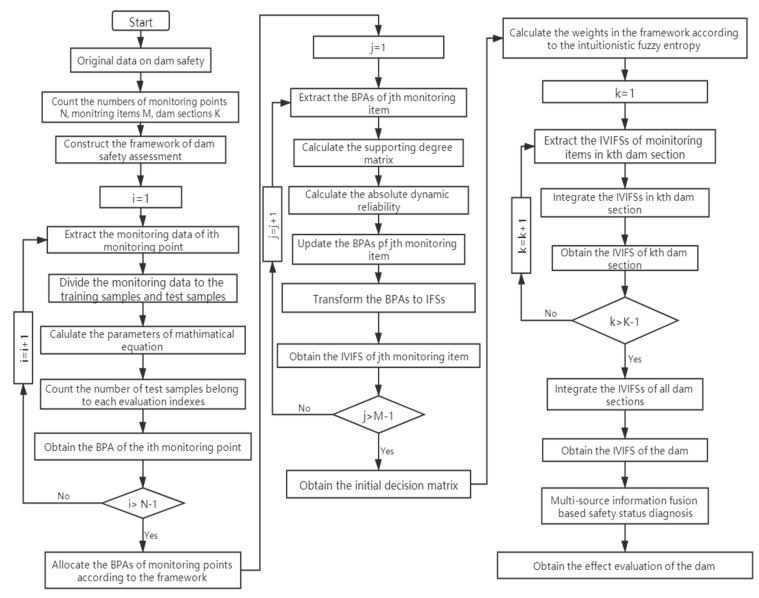
The algorithm flowchart of the dam safety assessment model.

To describe the methodology for practical implementation, the algorithm that implements the model is structured in Figure 5. From the flowchart in Figure 5, the framework of dam safety assessment in Figure 1 should be constructed firstly. According to the original data of the dam, the numbers of monitoring points, monitoring items, and dam sections can be obtained. Then the locations of monitoring points in the framework can be obtained according to their locations and usage. The locations of monitoring items in the framework can be obtained according to their usage. Therefore, the framework of the dam safety assessment can be obtained from the original data of the dam.

According to the framework in Figure 1, the monitoring point is located at the first-level model. Each monitoring point represents a monitoring sensor. To obtain the BPAs of monitoring points, the judgement criteria by trend performance is employed. The monitoring data is divided into training samples and test samples. The parameters in mathematical equation are obtained according to the training samples. The statistical result of the five evaluation indexes is obtained according to the test samples. The BPAs of monitoring points can be obtained.

Then the dynamic reliability analysis is employed to assess the reliability of each monitoring sensor. To modify the BPAs of monitoring points, the BPAs of each monitoring item are extracted. Then the supporting degree matrix and the dynamic reliability are calculated. The BPAs can be updated according to the dynamic reliability. To describe the uncertainty and fuzziness between different monitoring items, the BPAs of monitoring points at the first-level model are transformed into the IVIFS of each monitoring item at the second-level model. The initial decision matrix in the heterogeneous fusion process is obtained.

Finally, the IVIFSs of monitoring items at the second-level model are integrated into the IVIFS of the dam. According to the intuitionistic fuzzy entropy, the weights in the framework are calculated. Through the aggregation operation based on DST, the IVIFSs of monitoring items are integrated into IVIFSs of dam sections in the second fusion model. Then the IVIFSs of dam sections are integrated into the IVIFSs of the dam in the third fusion model. The fusion result of the dam can be obtained from the model. Through the safety status diagnosis, the potential risks and the effect evaluation of the dam can be obtained.

### 2.6. Criteria of the Assessment Performance for the Risk Assessment Model

To evaluate the assessment performance of the proposed model, the nearness degree between the ideal solutions is introduced. As mentioned above, the IVIFS of the dam are obtained in the third fusion model. The evaluation result of the dam can be presented as follows:

(39) $$IVIFS\;H=\left\{V_{j},\langle \mu^{l}\left(V_{j}\right),\mu^{u}\left(V_{j}\right)\rangle ,\langle \nu^{l}\left(V_{j}\right),\nu^{u}\left(V_{j}\right)\rangle |V_{j}\in V\right\}$$

Considering that the risks of all the monitoring points are different, the positive ideal solution can be served as the safest monitoring points that the membership degree of the first evaluation index is highest. The negative ideal solution can be served as the most dangerous monitoring points that the membership degree of the first evaluation index is lowest. Based on the analysis above, the IFSs of the positive and negative ideal solution can be presented as follows:

(40) $$\begin{array}{l} IFS{\;S}^{+}=IFS_{t^{+}},{\;[\max,t}^{+}]=\underset{j=1,\cdots ,N}{\max}\mu^{\prime }\left(V_{1}\right)\\ IFS{\;S}^{-}=IFS_{t^{-}},{\;[\min,t}^{-}]=\underset{j=1,\cdots ,N}{\min}\mu^{\prime }\left(V_{1}\right) \end{array}$$

where *N* is the number of the monitoring points, *t*^+^ is the ordinal number of the positive ideal solution, and *t*^−^ is the ordinal number of the negative ideal solution.

From the definition of IVIFS, the IFS can be served as a special case of the IVIFS. Therefore, the distance between IVIFSs can be employed. The nearness degree between the ideal solutions can be presented as follows:

(41) $$d=1-\frac{D\left(H,S^{+}\right)+D\left(H,S^{-}\right)}{2}$$

where *D*(*H*,*S*^+^) and *D*(*H*,*S*^−^) are the distances between the positive and negative ideal solutions.

## 3. Case Study

### 3.1. General Description of the Project

The project used as an example in this paper is a multiple-arch dam, which is situated on the Luo River in Anhui Province, China. The hydropower project officially began in 1950s. Because of the poor construction quality, the aging of the concrete, and corrosion of steel, by the 1990s there were many large cracks in the wall and serious water seepage in many places. Therefore, a large reinforcement project was implemented in 2002~2004. According to the dam risk assessment, the proposed multisource information fusion-based model is employed to evaluate the dam safety before and after the reinforcement.

### 3.2. Engineering Overview and Data Analysis

**Figure 6:**
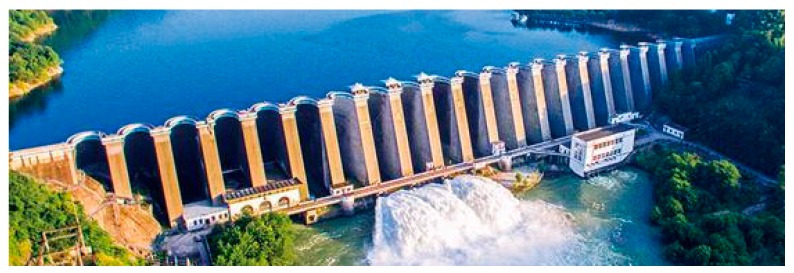
Layout of the multiple-arch dam.

**Figure 7:**
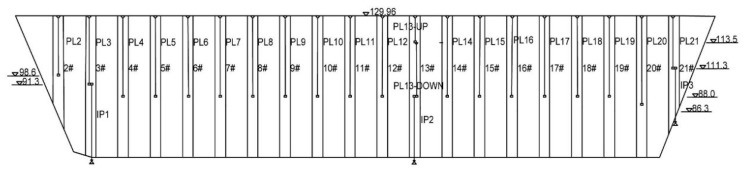
Pendulum systems for monitoring horizontal displacement.

The multiple-arch dam is mainly comprised by 20 partition walls and 21 arches showed in Figure 6. The maximum dam height is 75.9 m, and the dam crest length is 510 m. The maximum water level and minimum water level are 123.52 m, 102.3 m, respectively. To understand the real-time working status of the dam during operation, many kinds of sensors are installed in the dam. The aim is to assess the environmental and monitoring data for the dam behavior. Taking the monitoring sensors of horizontal displacement for example, there are 20 pendulum lines (PL) and three inverted pendulum lines (IP) in Figure 7. After the reinforcement implementation, many sensors are updated and the monitoring cycle has changed from three times a week to once a day.

**Figure 8:**
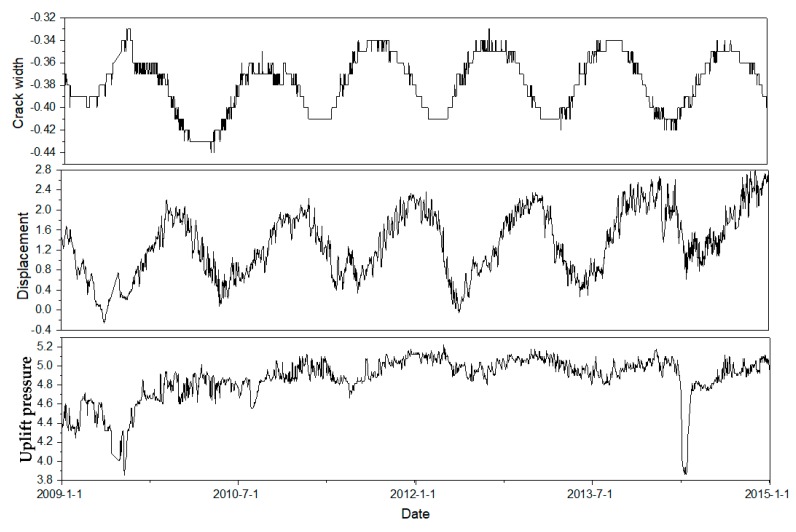
Displacement, seepage, and crack width recorded in the dam section #12.

Considering the severely missing data before 1998, time series with 646 data points from August 1999 to October 2003 are selected before reinforcement. As for the monitoring data after reinforcement, time series with 2371 data points from January 2009 to June 2015 are selected. Taking the dam section #12 for example, the time series of displacement, seepage, and crack width at certain monitoring point are presented in Figure 8.

All the time series are partitioned into training and test samples. The training samples are selected to calculate the parameter of the mathematical equation. The test samples are selected to evaluate the dam safety. To reflect the variation of evaluation results, there are five divisions for the time series before reinforcement in Table 2. A similar division goes for the time series after reinforcement.

Considering that there were many monitoring sensors only installed in a few dam sections and some sensors had broken down before reinforcement, only the dam sections #12~#14 are selected for this study. Because of the incomplete monitoring data for some monitoring items, only the width crack, vertical displacement, and uplift pressure are selected as the monitoring items.

### 3.3. The Evaluation Result of Monitoring Items

As mentioned above, there are many monitoring points for a monitoring item. To simplify the calculation process of the monitoring items, the width crack for dam section #12 is taken for example. There are five monitoring points for the width crack of the dam section #12.

**Figure 9:**
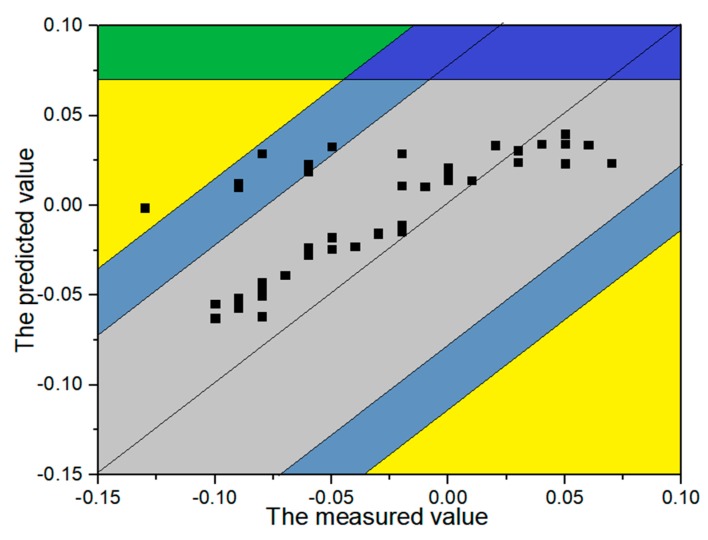
The risk gradation for the evaluation indexes at the first period.

According to the trend performance assessment, the mathematical equations of all monitoring points are obtained. Taking the monitoring point #j11 at the first period before reinforcement for example, the predicted and measured values of width crack are presented in Figure 9. According to the linear regression analysis between the measured and predicted values, the risk gradation for the evaluation indexes at the first period is presented in Figure 8. Counting the number of test samples located in the evaluation indexes, the statistical result of the five evaluation indexes for five monitoring points is presented in Table 3.

According to the statistical results of these evaluation indexes in Table 3, the basic probability assessment (BPA) of the monitoring point #j11 can be obtained with the Equation (3). Table 4 lists the basic probability assignment of monitoring point #j11. The intuitionistic fuzzy sets can be transformed from the BPAs by Equation (14). The intuitionistic fuzzy sets can be described as follows:

$$IFSs=\left\{\begin{array}{c} \langle V_{1},0.672,0.092\rangle ,\langle V_{2},0.084,0.068\rangle ,\langle V_{3},0.008,0.756\rangle ,\langle V_{4},0,0.764\rangle ,\langle V_{5},0,0.764\rangle \\ \langle V_{1},0.8,0\rangle ,\langle V_{2},0,0.8\rangle ,\langle V_{3},0,0.8\rangle ,\langle V_{4},0,0.8\rangle ,\langle V_{5},0,0.8\rangle \\ \langle V_{1},0.4,0.24\rangle ,\langle V_{2},0.12,0.52\rangle ,\langle V_{3},0.12,0.52\rangle ,\langle V_{4},0,0.64\rangle ,\langle V_{5},0,0.64\rangle \\ \langle V_{1},0.464,0.188\rangle ,\langle V_{2},0.06,0.592\rangle ,\langle V_{3},0.128,0.524\rangle ,\langle V_{4},0,0.652\rangle ,\langle V_{5},0,0.652\rangle \\ \langle V_{1},0.324,0.218\rangle ,\langle V_{2},0.108,0.434\rangle ,\langle V_{3},0.108,0.434\rangle ,\langle V_{4},0,0.542\rangle ,\langle V_{5},0,0.542\rangle \end{array}\right\}$$

The supporting degree matrix (SDM) for five BPAs is:

$$SDM=\left(\begin{array}{ccccc} 1 & 0.9441 & 0.8945 & 0.9187 & 0.8265\\ 0.9489 & 1 & 0.8441 & 0.8696 & 0.7766\\ 0.8631 & 0.8158 & 1 & 0.9691 & 0.9314\\ 0.8790 & 0.8354 & 0.9734 & 1 & 0.9070\\ 0.7586 & 0.7145 & 0.8958 & 0.8790 & 1 \end{array}\right)$$

Based on the Equation (18), the total supporting degrees can be calculated:

$$Total\_Sup\left(m_{j}\right)=\left\{3.4495,3.3099,3.6078,3.6363,3.4414\right\}$$

Then the dynamic reliabilities of monitoring points can be obtained according to the Equation (19):

$$R\left(m_{i}\right)=\left\{0.9486,0.9102,0.9921,1,0.9464\right\}$$

Based on the dynamic reliability, the original IFSs can be modified by the Equation (16). The interval-valued intuitionistic fuzzy set of the width crack of the dam section #12 can be expressed as follows:

$$IVIFS=\left(\begin{array}{l} \left\{V_{1},\left[0.212,0.7282\right],\left[0,0.2271\right]\right\}\\ \left\{V_{2},\left[0,0.1191\right],\left[0.324,0.8362\right]\right\}\\ \left\{V_{3},\left[0,0.1080\right],\left[0.324,0.8473\right]\right\}\\ \left\{V_{4},\left[0,0\right],\left[1,1\right]\right\}\\ \left\{V_{5},\left[0,0\right],\left[1,1\right]\right\} \end{array}\right)$$

From the evaluation result of the width crack for the dam section #12, it indicates that the dam section #12 remains normal at a higher level for the first period. Considering that there are many monitoring items involved in the risk assessment of the multiple-arch dam, other calculation data for other monitoring items are presented at the supplemental files.

### 3.4. The Fusion Process of the Dam Risk Assessment

According to the framework of dam risk assessment, the IVIFSs of monitoring items can be obtained in the first fusion model. Considering that the monitoring items are {*C*_1_, *C*_2_, *C*_3_} and the evaluation indexes are {*V*_1_, *V*_2_, *V*_3_, *V*_4_, *V*_5_}, the IVIFSs of monitoring items can be presented as follows:

$$D_{1}=\begin{array}{c} V_{1}\\ V_{2}\\ V_{3}\\ V_{4}\\ V_{5} \end{array}(\begin{array}{c} C_{1}\\ \left[0.324,0.7371\right],\left[0,0.2275\right]\\ \left[0,0.1195\right],\left[0.3240,0.8451\right]\\ \left[0,0.1080\right],\left[0.3240,0.8566\right]\\ \left[0,0\right],\left[1,1\right]\\ \left[0,0\right],\left[1,1\right] \end{array}\begin{array}{c} C_{2}\\ \left[0.368,0.664\right],\left[0.05,0.320\right]\\ \left[0.05,0.152\right],\left[0.368,0.832\right]\\ \left[0,0.168\right],\left[0.373,0.816\right]\\ \left[0,0\right],\left[1,1\right]\\ \left[0,0\right],\left[1,1\right] \end{array}\begin{array}{c} C_{3}\\ \left[0.208,0.347\right],\left[0.190,0.332\right]\\ \left[0.024,0.06\right],\left[0.374,0.619\right]\\ \left[0.166,0.272\right],\left[0.232,0.407\right]\\ \left[0,0\right],\left[1,1\right]\\ \left[0,0\right],\left[1,1\right] \end{array})$$

$$D_{2}=\begin{array}{c} V_{1}\\ V_{2}\\ V_{3}\\ V_{4}\\ V_{5} \end{array}(\begin{array}{c} C_{1}\\ \left[0.285,0.32\right],\left[0.215,0.434\right]\\ \left[0.096,0.202\right],\left[0.404,0.552\right]\\ \left[0.119,0.232\right],\left[0.381,0.522\right]\\ \left[0,0\right],\left[1,1\right]\\ \left[0,0\right],\left[1,1\right] \end{array}\begin{array}{c} C_{2}\\ \left[0.304,0.452\right],\left[0.288,0.424\right]\\ \left[0.120,0.200\right],\left[0.472,0.676\right]\\ \left[0.168,0.224\right],\left[0.424,0.652\right]\\ \left[0,0\right],\left[1,1\right]\\ \left[0,0\right],\left[1,1\right] \end{array}\begin{array}{c} C_{3}\\ \left[0.32,0.409\right],\left[0.165,0.275\right]\\ \left[0,0.035\right],\left[0.485,0.649\right]\\ \left[0.165,0.240\right],\left[0.320,0.444\right]\\ \left[0,0\right],\left[1,1\right]\\ \left[0,0\right],\left[1,1\right] \end{array})$$

$$D_{3}=\begin{array}{c} V_{1}\\ V_{2}\\ V_{3}\\ V_{4}\\ V_{5} \end{array}(\begin{array}{c} C_{1}\\ \left[0.320,0.368\right],\left[0.178,0.303\right]\\ \left[0.012,0.071\right],\left[0.456,0.600\right]\\ \left[0.166,0.232\right],\left[0.332,0.439\right]\\ \left[0,0\right],\left[1,1\right]\\ \left[0,0\right],\left[1,1\right] \end{array}\begin{array}{c} C_{2}\\ \left[0.335,0.397\right],\left[0.157,0.260\right]\\ \left[0,0.060\right],\left[0.492,0.597\right]\\ \left[0.157,0.200\right],\left[0.335,0.403\right]\\ \left[0,0\right],\left[1,1\right]\\ \left[0,0\right],\left[1,1\right] \end{array}\begin{array}{c} C_{3}\\ \left[0.208,0.432\right],\left[0.277,0.393\right]\\ \left[0.095,0.145\right],\left[0.390,0.680\right]\\ \left[0.182,0.248\right],\left[0.303,0.577\right]\\ \left[0,0\right],\left[1,1\right]\\ \left[0,0\right],\left[1,1\right] \end{array})$$

where {*C*_1_, *C*_2_, *C*_3_} represent the crack width, vertical displacement, and uplift pressure, respectively.

According to the intuitionistic fuzzy entropy, the comprehensive weight *ω* for monitoring items at the first time period can be calculated by Equation (25):

$$\omega =\left(\begin{array}{ccc} 0.439 & 0.420 & 0.141\\ 0.257 & 0.392 & 0.350\\ 0.322 & 0.386 & 0.292 \end{array}\;\right)$$

Then the IVIFSs of dam sections can be obtained by the fusion model based on DST:

$$E=\begin{array}{c} V_{1}\\ V_{2}\\ V_{3}\\ V_{4}\\ V_{5} \end{array}\left(\begin{array}{ccc} D_{1} & D_{2} & D_{3}\\ \left[0.136,0.452\right],\left[0.007,0.181\right] & \left[0.175,0.307\right],\left[0.079,0.243\right] & \left[0.232,0.462\right],\left[0.079,0.407\right]\\ \left[0.002,0.057\right],\left[0.140,0.540\right] & \left[0.009,0.054\right],\left[0.285,0.461\right] & \left[0.007,0.126\right],\left[0.339,0.723\right]\\ \left[0.004,0.087\right],\left[0.140,0.508\right] & \left[0.070,0.162\right],\left[0.187,0.380\right] & \left[0.072,0.234\right],\left[0.242,0.627\right]\\ \left[0,0\right],\left[1,1\right] & \left[0,0\right],\left[1,1\right] & \left[0,0\right],\left[1,1\right]\\ \left[0,0\right],\left[1,1\right] & \left[0,0\right],\left[1,1\right] & \left[0,0\right],\left[1,1\right] \end{array}\right)$$

The comprehensive weight *λ* of dam sections can be obtained by Equation (26):

λ=(03268,0.2669,0.4063)

According to the fusion model based on DST, the IVIFS of the dam at the first time period can be obtained:

$$H=\begin{array}{c} V_{1}\\ V_{2}\\ V_{3}\\ V_{4}\\ V_{5} \end{array}\left(\begin{array}{c} \left[0.083,0.620\right],\left[0.028,0.352\right]\\ \left[0.003,0.085\right],\left[0.111,0.701\right]\\ \left[0.025,0.205\right],\left[0.086,0.687\right]\\ \left[0,0\right],\left[1,1\right]\\ \left[0,0\right],\left[1,1\right] \end{array}\right)$$

The IVIFSs of the dam at the other time periods can be calculated using the fusion process mentioned above. The evaluation results are provided in the Supplementary Materials.

### 3.5. Comparison with other Conventional Decision Models

To illustrate the performance of the dam risk assessment model, the dam section #13 before reinforcement at the second time period is selected as a numerical example in this section.

In the dam section #13, there are eleven sensors *j*_1_ – *j*_11_ monitoring for crack width, vertical displacement, and uplift pressure. The BPAs of eleven monitoring points can be presented as follows:

$$C_{1}=\begin{array}{c} j_{1}\\ j_{2}\\ j_{3} \end{array}\left(\begin{array}{cccccc} V_{1} & V_{2} & V_{3} & V_{4} & V_{5} & \theta \\ 0.4 & 0.108 & 0.128 & 0 & 0 & 0.3664\\ 0.56 & 0.156 & 0.016 & 0 & 0 & 0.268\\ 0.712 & 0.024 & 0.008 & 0 & 0 & 0.256 \end{array}\right)$$

$$C_{2}=\begin{array}{c} j_{4}\\ j_{5}\\ \begin{array}{l} j_{6}\\ j_{7} \end{array} \end{array}\left(\begin{array}{cccccc} V_{1} & V_{2} & V_{3} & V_{4} & V_{5} & \theta \\ 0.48 & 0.12 & 0.08 & 0 & 0 & 0.32\\ 0.448 & 0.156 & 0.072 & 0 & 0 & 0.324\\ \begin{array}{l} 0.336\\ 0.448 \end{array} & \begin{array}{l} 0.12\\ 0.216 \end{array} & \begin{array}{l} 0.152\\ 0.024 \end{array} & \begin{array}{l} 0\\ 0 \end{array} & \begin{array}{l} 0\\ 0 \end{array} & \begin{array}{l} 0.392\\ 0.312 \end{array} \end{array}\right)$$

$$C_{3}=\begin{array}{c} j_{8}\\ j_{9}\\ \begin{array}{l} j_{10}\\ j_{11} \end{array} \end{array}\left(\begin{array}{cccccc} V_{1} & V_{2} & V_{3} & V_{4} & V_{5} & \theta \\ 0.4 & 0.108 & 0.128 & 0 & 0 & 0.364\\ 0.704 & 0.072 & 0 & 0 & 0 & 0.224\\ \begin{array}{l} 0.352\\ 0.464 \end{array} & \begin{array}{l} 0.062\\ 0.12 \end{array} & \begin{array}{l} 0.166\\ 0.088 \end{array} & \begin{array}{l} 0\\ 0 \end{array} & \begin{array}{l} 0\\ 0 \end{array} & \begin{array}{l} 0.42\\ 0.328 \end{array} \end{array}\right)$$

where {*C*_1_, *C*_2_, *C*_3_} represent the crack width, vertical displacement, and uplift pressure, respectively.

Table 5 shows the fusion results obtained with the other different methods. From Table 5, the dam section #13 remains normal at the second time through all the conventional decision model. The possibility of falling into the first evaluation index is up to 0.99 through the Classical Dempster’s rule. Taking the vertical displacement for example, the fusion results show that the possibility of the second and third evaluation indexes almost decrease to 0.04. However, the possibility of falling into the second and third evaluation indexes is high according to the BPAs of four monitoring sensors *j*_4_~*j*_7_. Such behavior shows that DST is risky to ignore the possibility of falling into other evaluation indexes under the fact that the possibility of unknown degree is high.

Once the dynamic reliability analysis is applied to the fusion process, the fusion results become reasonable. Taking the vertical displacement for example, the possibility of falling into the second and third evaluation degree is up to 0.28, which is similar to the BPAs of four monitoring sensors *j*_4_~*j*_7_. However, the fusion result of the dam section #13 is unreasonable. The possibility of the first evaluation is up to 0.94 through the Dempster’s rule, which indicates that DST is risky to increase the possibility of falling into the first evaluation index through multiple fusions. Therefore, it is unreasonable to integrate the information just by DST.

The fusion result of IVIFS and DST show that the dam section #13 remains normal during the second time period and the possibility of falling into other evaluation indexes is up to 0.2. Compared with the decision model mentioned above, the fusion result is more reasonable. However, only the possibility of the first evaluation index is up to 0.7 from the monitoring sensors *j*_3_ and *j*_9_ and all the possibilities from other monitoring sensors do not exceed 0.6, which indicates that the fusion result is greatly influenced by monitoring sensors *j*_3_ and *j*_9_. The BPAs of two monitoring sensors are just special cases, which do not show similar distribution to those of other monitoring sensors. Therefore, it is suitable to modify the BPAs of all monitoring sensors by dynamic reliability analysis.

From the final result of the proposed model, the weight of *C*_2_ is 0.419. From the BPAs of all monitoring sensors, the monitoring sensors except *j*_3_ and *j*_9_ have similar performance. Compared with the fusion result of IVIFS & DS, the weight of *C*_1_ decreases from 0.387 to 0.239. This indicates that the weights of abnormal monitoring sensors are low and the weights of these monitoring sensors with similar performance are high after the dynamic reliability analysis is employed.

To describe the assessment performance of the proposed model clearly, the nearness degrees of all the models are showed in Table 6. In Table 6, the distances between the ideal solutions become smaller after transforming the BPAs to IVIFSs. It indicates that the fusion results of conventional decision models are far from the ideal solutions and are closer to the positive ideal solution. But the positive ideal solution is just a special case according to the BPAs of these monitoring points. Therefore, the fusion results of the conventional decision model are influenced by the positive ideal solution and ignore the BPAs of other monitoring points. It is suitable to transform the BPAs to IVIFSs. Comparison between four decision models, the nearness degree of the proposed model is highest. It indicates that the proposed model performs well in the assessment performance.

Above all, the fusion result of the proposed model is more reasonable than that provided by other different models even if two abnormal monitoring sensors perform differently from other monitoring sensors. The possibility of falling into the second and third is not ignored through the fusion process, so the proposed model can provide more reasonable and helpful results for multiple-source information fusion.

## 4. Results and Discussion

### 4.1. The Safety Evaluation Results before Reinforcement

The IVIFSs of the dam for the five time periods is presented as follow. Considering that the BPAs of the fourth and fifth evaluation indexes are lower than 10^−5^, the IVIFSs only provide the first to third evaluation indexes:

$$H=\begin{array}{c} T_{1}\\ T_{2}\\ T_{3}\\ T_{4}\\ T_{5} \end{array}\left(\begin{array}{ccc} V_{1} & V_{2} & V_{3}\\ \left[0.083,0.620\right],\left[0.028,0.352\right] & \left[0.003,0.085\right],\left[0.111,0.701\right] & \left[0.025,0.205\right],\left[0.086,0.687\right]\\ \left[0.079,0.884\right],\left[0.006,0.259\right] & \left[0.002,0.105\right],\left[0.083,0.844\right] & \left[0.003,0.092\right],\left[0.081,0.846\right]\\ \left[0.092,0.757\right],\left[0.003,0.132\right] & \left[0.002,0.071\right],\left[0.093,0.761\right] & \left[0,0.044\right],\left[0.095,0.749\right]\\ \left[0.125,0.769\right],\left[0.005,0.207\right] & \left[0.005,0.117\right],\left[0.126,0.790\right] & \left[0,0.060\right],\left[0.131,0.785\right]\\ \left[0.144,0.570\right],\left[0.003,0.064\right] & \left[0.003,0.053\right],\left[0.144,0.573\right] & \left[0,0.092\right],\left[0.148,0.585\right] \end{array}\right)$$

From the fusion result, it shows that the dam remains normal at the five time periods. But the total BPAs of the second and third evaluation indexes reaches 0.15, which indicates that the dam have higher possibility to transform from the first evaluation index to the second or third indexes. The distances between the IVIFSs for the five time periods and the positive ideal solutions are presented in Table 7.

**Figure 10:**
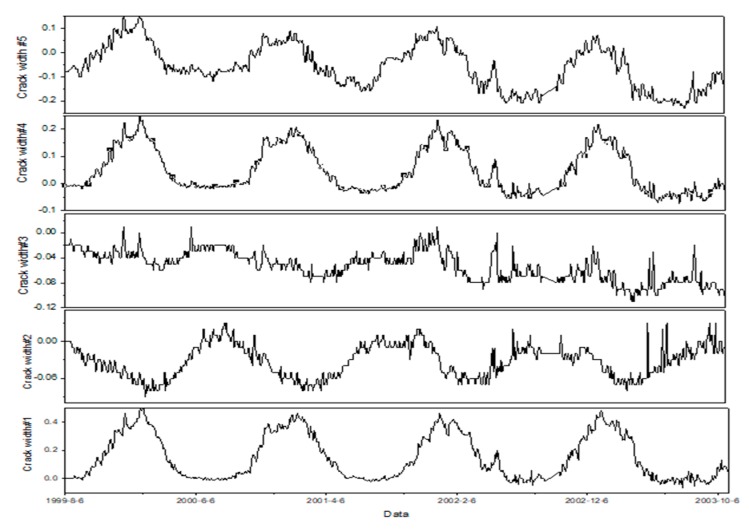
The variation of crack width for five monitoring points at dam section #12.

Table 7 indicates that the distances are changing as the time goes by. To illustrate the variation of dam safety, the width crack of dam section #12 is taken for example. Figure 9 shows the variation of crack width for five monitoring points. The width crack remains at lower value area and the dam become safer at the second time period in Figure 10. From the Table 7, the distance between the ideal solution of *V*_1_ becomes closer and the distance between that of *V*_3_ becomes further at the second time period. Then the crack width remains at a higher value level and there exist peaks at the third time period. Therefore, the distances between the positive ideal solution of $V_{1}$~$V_{3}$ become further. It indicates the dam behavior is changing to the fourth evaluation index at the third time period. From the analysis above, the distances between IVIFS and positive ideal solutions can describe the variation of dam safety.

As mentioned above, the total BPAs of the second and third evaluation indexes reach 0.15. This indicates that the dam will fall into the second and third evaluation indexes. If the dam remains normal all the time, the distance between the positive ideal solution of *V*_1_ will be smaller. Therefore, there exists potential risk of the dam. To identify the potential risk of the dam, the weight variations of dam sections are employed in this section. Table 8 shows the weight variation of three dam section at five time periods.

Compared with the other dam sections, the weights of the dam section #13 are lowest at the five time periods in Table 8. It shows that the dam section #13 lowers the safety of the whole multiple-arch dam. Table 9 shows the weight of three monitoring items at the five time periods. The weights of the uplift pressure are lower than other monitoring items. The weights of uplift pressure at five time periods are as low as 0.25. Table 10 shows the basic probability assessment of monitoring point #1 for uplift pressure. From the BPAs of monitoring point #1, the probability of dam section #13 falling into the second and third evaluation indexes is as high as 30%. Therefore, something serious were more likely to happen in the dam section #13. According to the geological survey before reinforcement, there existed weak fracture zone and broken mud belt in the foundation of the dam section #13. Therefore, the foundation of dam section #13 was reinforced at 2002~2004.

### 4.2. The Safety Evaluation Results after Reinforcement

Because of the reinforcement, the multiple-arch dam became safer than before. The IVIFSs of the dam at five time periods after reinforcement is presented as follows:

$$H=\begin{array}{c} T_{1}\\ T_{2}\\ T_{3}\\ T_{4}\\ T_{5} \end{array}\left(\begin{array}{cc} V_{1} & V_{2}\\ \left[0.361,0.900\right],\left[0.002,0.030\right] & \left[0.002,0.030\right],\left[0.361,0.900\right]\\ \left[0.409,0.871\right],\left[0.006,0.020\right] & \left[0.006,0.020\right],\left[0.409,0.871\right]\\ \left[0.295,0.863\right],\left[0.001,0.028\right] & \left[0.001,0.028\right],\left[0.295,0.863\right]\\ \left[0.320,0.865\right],\left[0.001,0.027\right] & \left[0.001,0.027\right],\left[0.320,0.865\right]\\ \left[0.310,0.854\right],\left[0.003,0.037\right] & \left[0.003,0.037\right],\left[0.310,0.854\right] \end{array}\right)$$

where *H* is the IVIFSs of the dam after reinforcement. According to the statistical results of all monitoring points, all the calculated data belongs to the first and second evaluation indexes. The IVIFSs only provide the first and second evaluation indexes.

From the evaluation result, the multiple-arch dam remained stably normal at all the five time periods. Table 11 shows the weights of all the dam sections at five time periods. The weights of all the dam section are similar at all five time periods. It indicates that all the dam sections have the similar performance and all sections of the dam hold the same importance at the safety assessment. Taking dam section #12 for example, Table 12 shows the weights of three monitoring items at five time periods. The weights of three monitoring items are similar at all five time periods. Therefore, the multiple-arch dam remains normal and stable after reinforcement.

### 4.3. Safety Comparison before and after Reinforcement

As mentioned above, a large reinforcement project was implemented in 2002~2004. To verify the feasibility of the dam safety assessment model, a comprehensive comparison is made between the evaluation results before and after reinforcement. Table 13 shows the distances between the IVIFSs of first evaluation index and the ideal solution. The distances have decreased from 0.6485 to 0.3693, which indicates that the safety degree of the dam has increased. The distance variation became smaller after reinforcement. It indicates that the dam became more stable after reinforcement.

To analyse the distance variation, the variation of dam safety is presented at Table 14. Considering that the BPAs of the fourth and fifth evaluation indexes is lower than 10^−5^, only the other evaluation indexes are considered in Table 14. In the Table 14, X,Y,Z represent the distances between the IVIFSs and the ideal solution of three evaluation indexes. Taking the ideal solution of the first evaluation index for example, it represents that the dam remains absolutely normal. Therefore, (0,1,1), (1,0,1) and (1,1,0) represent the positions of ideal solutions of three evaluation indexes, respectively. From Table 14, the safety degree before reinforcement is closer to the ideal solution of the first evaluation index. The variation range of distances is large and tends to become larger. It indicates that the dam has the tendency to falling into the worse situation before reinforcement. Compared with the variation before reinforcement, the safety degree after reinforcement becomes closer to the ideal solution of the first evaluation index and the variation range becomes smaller. It indicates that the dam becomes safer and more stable after reinforcement. Therefore, the dam behavior becomes normal after reinforcement and tends to remain normal at a larger period.

From the comparison between the fusion results at different time series, the proposed model can be used to analyze the dam safety according to the dam behavior. Through the analysis to the distance variation and the weights of monitoring items, the potential risk location can be identified. Therefore, it is suitable to assess the dam safety through the multisource fusion-based model.

## 5. Conclusions

In this paper, a new multisource fusion model based on interval-valued intuitionistic fuzzy sets and evidence theory is employed to assess dam safety. Considering the homologous information obtained from different monitoring points, interval-valued intuitionistic fuzzy sets are used to describe the variation of homologous information. For the heterogeneous information obtained from different monitoring items and different dam sections, evidence theory is employed to extract the features and integrate the IVIFSs. For verification, the dam safety assessment model is used to obtain the evaluation results for a multiple-arch dam before and after reinforcement. From the fusion results, the multiple-arch dam becomes safer and more stable after reinforcement. Through the analysis for the evaluation results, the potential risk is likely to be located at the dam section #13, which goes same with the result of geological survey for the multiple-arch dam. Therefore, the dam safety assessment model performs well in evaluating the dam safety and identifying the potential risk location. Generally, the major contributions of this study of the dam safety assessment are highlighted as follows:

(1) Considering the difference between homologous information, the dynamic reliability based on supporting degree is employed to extract the common feature and modifying the BPAs. To reflect the variation of BPAs from different monitoring points, the interval-valued intuitionistic fuzzy set is used to describe the variation. Therefore, the IVIFSs can consider the similarities and differences between different monitoring points. From the comparison with other decision models, the proposed model can provide more reasonable and helpful result for multi-source information fusion.
(2) To reflect the difference between heterogeneous information, the intuitionistic fuzzy entropy is used to obtain the objective weights of different monitoring items and dam sections. From the analysis with the weights of different dam sections, the potential risk is located at the dam section #13. Therefore, the performance of dam structural behavior can be evaluated through the proposed model. The potential risk can be identified through analysis with the weight of all the influencing factors. With a large number of dams entering an advanced age, regular reinforcement measures should be adopted in the future. The proposed model can be used to diagnose accurately the dam behavior, obtain the safety degree, and evaluate potential risks for proper reinforcement.
(3) From the analysis with evaluation results at different time periods, the dam behavior becomes normal and stable after reinforcement. The effect of reinforcement measures on improving dam behavior is verified.

However, there exist several deficiencies in the proposed model. The deficiencies of the proposed model and the direction of future researches are pointed out. Firstly, the transformation from BPAs to IVIFS in Equation (2) only takes the maximum and minimum value into consideration, and ignores the information in the interval. Secondly, the weights of monitoring indexes are only determined based on the intuitionistic fuzzy entropy. However, the weights are influenced by dam type, the number of sensors, the distribution of sensors, etc. It is appropriate to consider all these factors. Thirdly, the weights in the proposed model are precise numbers. The dam performs differently in each time period. If the weights are interval number obtained from all the time periods, the fusion result will become more reasonable.

## Figures and Tables

**Table 1:** The evaluation index for single monitoring point. Where *y* is the measured value of testing samples, $\bar{y}$ is the predicted value of testing samples, *S* are the mean square deviations of the difference between the calculated and measured values: $S=\sum_{i=1}^{n}{\left(y_{i}-{\bar{y}}_{i}\right)}^{2}/n$.

Evaluation Index	Measurement Standard
Normal (V_1_)	$y\leq y_{\max},\left|y-\bar{y}\right|<2S$
Nearly normal (V_2_)	$y\leq y_{\max},2S<\left|y-\bar{y}\right|<3S$
Mildly normal (V_3_)	$y\leq y_{\max},y>\left|\bar{y}+3S\right|$
Severely abnormal (V_4)_	$y>y_{\max},y\leq \left|\bar{y}+3S\right|$
Malignant abnormal (V_5_)	$y>y_{\max},y>\left|\bar{y}+3S\right|$

**Table 2:** Five division for the time series before and after reinforcement.

Before Reinforcement Implementation		After Reinforcement Implementation	
Training Time Series	Testing Time Series	Training Time Series	Testing Time Series
1999.8–2000.12	2000.12–2001.3	2009.1–2012.11	2012.11–2013.2
1999.8–2001.7	2001.7–2001.11	2009.1–2013.5	2013.5–2013.8
1999.8–2002.3	2002.3–2002.6	2009.1–2013.12	2013.12–2014.3
1999.8–2002.11	2002.11–2003.3	2009.1–2014.6	2014.6–2014.10
1999.8–2003.7	2003.7–2003.11	2009.1–2015.2	2015.2–2015.6

**Table 3:** The statistical number of the five evaluation indexes at the first period.

Monitoring Points	V_1_	V_2_	V_3_	V_4_	V_5_
**1**	42	7	1	0	0
**2**	50	0	0	0	0
**3**	25	10	15	0	0
**4**	29	5	16	0	0
**5**	14	9	26	0	0

**Table 4:** The BPAs of the five monitoring points for the first period.

Monitoring Points	V_1_	V_2_	V_3_	V_4_	V_5_
**1**	0.672	0.084	0.008	0	0
**2**	0.8	0	0	0	0
**3**	0.4	0.12	0.12	0	0
**4**	0.464	0.06	0.128	0	0
**5**	0.224	0.108	0.208	0	0

**Table 5:** The fusion results of different decision models.

		*C*_1_	*C*_2_	*C*_3_	*E*
Classical Dempster’s rule	$V_{1}$	0.910	0.818	0.904	1.000
	$V_{2}$	0.042	0.110	0.034	0
	$V_{3}$	0.016	0.040	0.038	0
	Θ	0.032	0.032	0.024	0
Dynamic reliability analysis & DST	$V_{1}$	0.847	0.668	0.797	0.941
	$V_{2}$	0.075	0.181	0.085	0.011
	$V_{3}$	0.043	0.110	0.088	0.010
	Θ	0.035	0.041	0.031	0.038
IVIFS & DST	$V_{1}$	[0.4,0.712]	[0.336,0.480]	[0.352,0.704]	[0.139,0.773]
	$V_{2}$	[0.024,0.156]	[0.120,0.216]	[0.062,0.120]	[0.013,0.109]
	$V_{3}$	[0.008,0.128]	[0.080,0.152]	[0,0.166]	[0.002,0.107]
	ω	0.387	0.249	0.364	
Proposed model	$V_{1}$	[0.4,0.702]	[0.335,0.478]	[0.350,0.649]	[0.150,0.676]
	$V_{2}$	[0.022,0.156]	[0.120,0.214]	[0.012,0.119]	[0.015,0.141]
	$V_{3}$	[0.006,0.128]	[0.024,0.152]	[0,0.215]	[0.013,0.164]
	ω	0.239	0.419	0.342	

**Table 6:** The nearness degree of different decision models.

	*D*(*H,S*^+^)	*D*(*H,S*^−^)	*d*
Classical Dempster’s rule	0.0896	0.2264	0.842
Dynamic reliability analysis & DST	0.0702	0.2066	0.8616
IVIFS & DST	0.1253	0.1364	0.8692
Proposed model	0.1205	0.1246	0.8775

**Table 7:** The distance between IVIFSs and the positive ideal solution.

Evaluation Index	T_1_	T_2_	T_3_	T_4_	T_5_
Normal	0.649	0.518	0.575	0.553	0.643
Nearly normal	0.956	0.947	0.963	0.939	0.972
Mildly normal	0.885	0.952	0.978	0.970	0.996

**Table 8:** The weight variation of three dam section at five time periods.

Dam Section	T_1_	T_2_	T_3_	T_4_	T_5_
#12	0.3268	0.4336	0.4233	0.3344	0.3353
#13	0.2669	0.2373	0.2749	0.3424	0.2704
#14	0.4063	0.3291	0.3018	0.3561	0.3943

**Table 9:** The weight variation of monitoring items for the dam section #13.

Monitoring Items	T_1_	T_2_	T_3_	T_4_	T_5_
Crack width	0.3503	0.4421	0.3424	0.4960	0.4513
Vertical displacement	0.3922	0.3189	0.3786	0.2157	0.3926
Uplift pressure	0.2575	0.2391	0.2789	0.2883	0.1561

**Table 10:** The BPAs of monitoring point #1 at five time periods.

Time Periods	V_1_	V_2_	V_3_	V_4_	V_5_
1	0.32	0.12	0.232	0	0
2	0.367	0.096	0.151	0	0
3	0.285	0.201	0.119	0	0
4	0.285	0.12	0.119	0	0
5	0.367	0.202	0.232	0	0

**Table 11:** The weights of all the dam sections at five time periods.

Dam Section	T_1_	T_2_	T_3_	T_4_	T_5_
#12	0.3170	0.3418	0.3430	0.3539	0.3145
#13	0.3236	0.3248	0.3258	0.3151	0.3409
#14	0.3595	0.3334	0.3312	0.3310	0.3446

**Table 12:** The weights of all the monitoring items at five time periods for dam section #12.

Monitoring Item	T_1_	T_2_	T_3_	T_4_	T_5_
Crack width	0.3346	0.3447	0.3367	0.3367	0.3432
Vertical displacement	0.3419	0.3385	0.3375	0.3404	0.3250
Uplift pressure	0.3235	0.3168	0.3258	0.3229	0.3318

**Table 13:** The distance between the IVIFSs of first evaluation index and the ideal solution.

Time Period	T_1_	T_2_	T_3_	T_4_	T_5_
Before reinforcement	0.6485	0.5185	0.5753	0.5526	0.6428
After reinforcement	0.3693	0.3596	0.4207	0.4075	0.4180

**Table 14:** The distance between the IVIFSs and the ideal solution before and after reinforcement.

Time Period	Before Reinforcement			After Reinforcement		
	X	Y	Z	X	Y	Z
T_1_	0.419	0.681	0.636	0.193	0.810	1
T_2_	0.288	0.705	0.708	0.186	0.813	1
T_3_	0.321	0.695	0.700	0.218	0.782	1
T_4_	0.329	0.698	0.714	0.211	0.789	1
T_5_	0.338	0.665	0.681	0.219	0.781	1

## Supplementary Materials

The following are available online at https://www.mdpi.com/1424-8220/20/9/2648/s1.
